# Improving the accuracy of the *k*-shell method by removing redundant links: From a perspective of spreading dynamics

**DOI:** 10.1038/srep13172

**Published:** 2015-08-17

**Authors:** Ying Liu, Ming Tang, Tao Zhou, Younghae Do

**Affiliations:** 1 Web Sciences Center, University of Electronic Science and Technology of China, Chengdu 610054, China; 2 School of Computer Science, Southwest Petroleum University, Chengdu 610500, China; 3 Section for Science of Complex Systems, Medical University of Vienna, Vienna 1090, Austria; 4 Big Data Research Center, University of Electronic Science and Technology of China, Chengdu 610054, China; 5 Department of Mathematics, Kyungpook National University, Daegu 702-701, South Korea

## Abstract

Recent study shows that the accuracy of the *k*-shell method in determining node coreness in a spreading process is largely impacted due to the existence of core-like group, which has a large *k*-shell index but a low spreading efficiency. Based on the analysis of the structure of core-like groups in real-world networks, we discover that nodes in the core-like group are mutually densely connected with very few out-leaving links from the group. By defining a measure of diffusion importance for each edge based on the number of out-leaving links of its both ends, we are able to identify redundant links in the spreading process, which have a relatively low diffusion importance but lead to form the locally densely connected core-like group. After filtering out the redundant links and applying the *k*-shell method to the residual network, we obtain a renewed coreness *k*_*s*_ for each node which is a more accurate index to indicate its location importance and spreading influence in the original network. Moreover, we find that the performance of the ranking algorithms based on the renewed coreness are also greatly enhanced. Our findings help to more accurately decompose the network core structure and identify influential nodes in spreading processes.

The development of network science has made it a powerful tool to model and analyze complex systems in nature and society^1^. One fundamental aspect is to understand the complex structures and behaviors of real-world networks^2^,^3^,^4^. Network structure can be described from the local, global and meso-scale levels^5^ such as node degree, clustering, degree distributions, degree correlations, motifs, communities, hierarchies, etc. The *k*-shell decomposition is a method used to partition a network into hierarchically ordered sub-structures^6^. It decomposes a network in an iterative way, removing all nodes of degree less than current shell index until no removing is possible (see Methods for details). Each node is assigned an index *k*_*s*_ to represent its coreness in the network. Nodes with the same *k*_*s*_ constitute the *k*_*s*_-shell. A large *k*_*s*_ indicates a core position in the network, while a small *k*_*s*_ defines the periphery of the network. The *k*-core, nodes with *k*_*s*_ ≥ *k*, obtained in the decomposition process is a highly interconnected substructure in network topology^7^, which has found its application in different fields of science, like biology^8^,^9^, economics^10^, and social science^11^,^12^,^13^,^14^. For example, nodes in the inner core (large *k*_*s*_ region) have a relatively high probability of being essential and evolutionary conserved in the protein interaction network^8^. Nodes in the innermost core (the shell with the largest *k*_*s*_ value in the network) of the global economic network are most probable to trigger out an economic crisis^10^. High *k*-cores of the air transportation network in USA are extremely resilient to both the node removal and edge removal^11^. Because of its low computational complexity of *O*(*N* + *E*)^15^, where *N* is the network size and *E* is the number of edges in the network, the *k*-shell method is extensively used in analyzing the hierarchical structure of large-scale networks, such as visualizing networks^16^, depicting the network core-periphery features^17^,^18^, and analyzing the Internet and its core^19^,^20^,^21^. In addition, the *k*-core is used to construct network model^22^, applied in community detection^23^ and *k*-core percolation is extensively studied which gives a notion of network resilience under random attack^24^,^25^. The *k*-shell method is also extended to weighted networks^26^, dynamic networks^27^ and multiplex networks^28^.

Considering that the *k*-shell method decomposes the network into ordered shells from the core to the periphery, researchers found that core nodes of the network are more influential than the periphery nodes in a spreading dynamics^29^. Following the work, there is growing interest in using the *k*_*s*_ index to rank nodes of their spreading efficiency. Nodes with large *k*_*s*_ are considered to be more influential and effective than others in a spreading process^10^,^30^,^31^,^32^. Furthermore, some works devise ranking algorithms based on *k*_*s*_ of nodes^33^,^34^,^35^. Despite its effectiveness, however, the coreness determined by the *k*-shell method has some limitations in identifying influential spreaders. In the rumor spreading model, nodes with high coreness are not influential spreaders but act as firewall to prevent the rumor from spreading to the whole network^36^. For dynamics with steady state, nodes with the highest degree act more importantly than the core nodes in uncorrelated networks if the degree distribution of the network has a decay exponent larger than 5/2^37^. In network with tree structure or BA model network, most of the nodes are assigned a same *k*_*s*_ value, thus the *k*-shell index is unable to distinguish node importance^38^. In particular, in our recent study^39^ we show that in some real-world networks the core nodes as identified by the *k*-shell decomposition are not the most influential spreaders. Specifically speaking, there exist core-like groups which are identified as cores with large *k*_*s*_ but are in fact only locally densely connected groups with relatively low spreading efficiency. This implies that the *k*_*s*_ index may be inaccurate to reflect the location importance of nodes in networks with such local structure, which proposes a great challenge for works using the *k*-shell method to identify network cores and rank nodes.

In this paper, we explore the topological feature of the core-like groups and find out the connection pattern that causes the failure of the *k*_*s*_ index to accurately determine the location importance and spreading influence of nodes in networks with such local structure. Furthermore, we propose a way to improve the accuracy of the *k*-shell method in determining node coreness from the perspective of spreading dynamics. Motivated by the research advances in core-periphery structure^40^,^41^,^42^, in which core nodes are not only densely connected among themselves but also well connected to the periphery nodes, which are sparsely connected to any other, we consider the characteristics of links a core node should have. Specifically speaking, links of core nodes should not only connect to core nodes, but also connect to nodes that are not in the core. To quantitatively determine the effect of a link in a spreading process, we define a measure of diffusion importance based on the connection patterns of its two ends. We find that there exist some redundant links in real-world networks, which have a low diffusion importance but lead to form the core-like group. By filtering out the redundant links from the original network and applying the *k*-shell decomposition on the residual network, we obtain a renewed coreness *k*_*s*_ for each node. This *k*_*s*_ is a much more accurate index to indicate the node importance in a spreading dynamics in the original network. We validate this by simulating the susceptible-infected-recovered (SIR) epidemic process on networks and comparing the spreading efficiency of nodes from the core to the periphery, which is used in many research works^29^,^31^. Furthermore, we find that ranking algorithms based on the *k*-shell method are also greatly enhanced once using the renewed *k*_*s*_ obtained from the residual network.

## Results

We first present the structural feature of the locally densely connected groups that cause the inaccuracy of the *k*-shell method in determining coreness of nodes in a spreading dynamics. We then define the diffusion importance of edges and remove the redundant edges. Finally, we validate the improved accuracy of the renewed coreness from the perspective of spreading dynamics.

### Structural feature of the locally densely connected group

We first focus on six real-world networks in which the *k*-shell method fails to identify the core shells because of the existence of the core-like groups^39^ (For the identification of core-like groups, see Methods for details). The properties of the studied networks are listed in Table 1.

**Figure 1:**
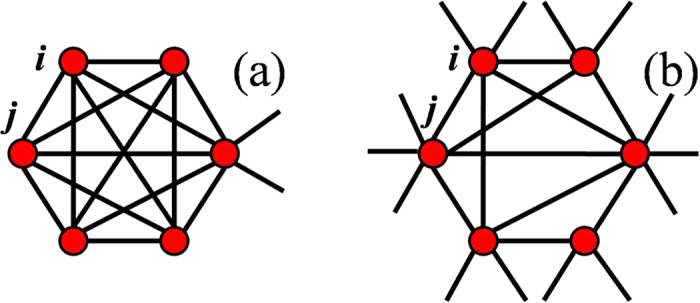
Illustration of structural feature of the core-like group and the true core. (**a**) Core-like group. (**b**) True core. For the core-like group, core nodes are mutually connected and have very few out-leaving links. While for the true core, core nodes are connected and each of them has a lot of out-leaving links.

Based on in-depth analysis of the network local structure, we find that the core-like group has a clique-like local structure as shown in Fig. 1(a). Most of the nodes in the core-like group have a similar connection pattern. Let’s take node *i* for example. Neighbors of node *i* are mutually connected, with only one neighbor having a few out-leaving links, that are links connecting outside the neighborhood of node *i*. In the *k*-shell decomposing process, node *i* will be assigned a *k*_*s*_ value equal to its degree. Considering the feature of core in the core-periphery structure^40^,^42^, which is densely connected among themselves and well connected to the periphery, we think that the cohesive group shown in Fig. 1(a) is not a true core, because it is only densely connected within a group but not well connected to the remaining part of the network. When a disease origins from node *i*, most of the infections are limited in the neighborhood of node *i*. As for the true core in Fig. 1(b), core nodes are well connected and at the same time connect well to the outside of the core. When a disease or rumor origins from node *i*, it is easier to spread to a broad area of the network through neighbors of node *i* whose links are connecting to the external parts of *i*'s neighborhood. We take the innermost core of the network CA-Hep and Router for example and visualize the connection pattern of the innermost core by the software Gephi of version 0.8.2^43^. We find that for the innermost core of CA-Hep (core-like group), which is the 31-shell composed of 32 mutually connected nodes, has a structure very similar to the structure shown in Fig. 1(a), with only five nodes having a small number of links out leaving the group, as shown in Fig. S1 (a) in Supporting Information (SI). As for the innermost core of Router (true-core), which is the 7-shell composed of 26 nodes, each node connects to a large amount of nodes that are not in the core-shell, as shown in Fig. S1 (b). Motivated by the structural difference of the core-like group and the true core, we think that the importance of links of a node *i* varies depending on the connection pattern of its neighbor nodes (e.g. node *j*): if node *j* has many connections out-leaving node *i*'s neighborhood, the probability of infecting more nodes increases when the spreading origins from node *i*, and thus the edge linking node *i* and node *j* is important for node *i*. On the contrary, if node *j* has very few or even no out-leaving links from node *i*'s neighborhood, the probability of infecting a large population by node *i* decreases, and thus the edge linking node *i* and node *j* is less important.

**Figure 2:**
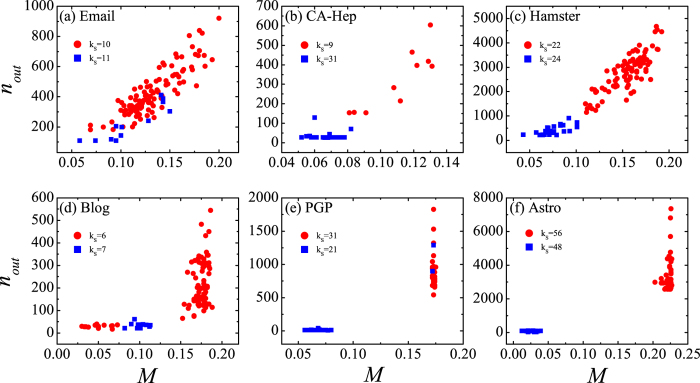
Correlation of spreading efficiency and the number of out-leaving links. For each network, we present the nodes in the core-like group (blue squares) and in the highest spreading efficiency shell (red circles). A positive correlation between the spreading efficiency and the number of out-leaving links is demonstrated.

To confirm the relationship between the structural feature and spreading behavior of the network, we use the SIR spreading model^44^ to simulate the spreading process on networks. We record the spreading efficiency of each node, which is the size of the final infected population *M* when a spreading origins from the node (see Methods for details). Then we study the correlation between the total number of out-leaving links *n*_*out*_ of a node, that is the sum of out-leaving links over all neighbors of the node, and its spreading efficiency *M*. To compare the difference between the core-like group and the true core, we choose two groups of nodes for each network. The first one is the shell that is a core-like group (there may be several core-like groups in the network, and we choose the one with the largest *k*_*s*_ value); the second one is the shell with the highest average spreading efficiency. From Fig. 2 we can see that in general nodes in core-like groups (blue squares), which have a relatively low spreading efficiency, have a lower number of out-leaving links than nodes in the highest spreading efficiency shell (red circles). What is worth noticing is that although most nodes in core-like groups have a relatively low spreading efficiency, there may be some nodes that have a high spreading efficiency, corresponding to some blue squares in Email and PGP, which also have a relatively high number of out-leaving links. On the other hand, in the highest spreading efficiency shell, there are nodes with relatively low spreading efficiency whose number of out-leaving links is correspondingly low, such as some red circles in Email and Blog. These indicate a positive correlation between the spreading efficiency and the number of out-leaving links of a node through its neighbors.

Considering the structural feature of core-like groups and the correlation between the number of out-leaving links and the spreading efficiency of a node, we realize that in the locally densely connected structures, there exist some links which lead to form a clique-like local structure but contribute little to the spreading process. This causes the failure of *k*-shell method in accurately determining node coreness and identifying true cores in many real-world networks in a spreading dynamics. Next we will step further to find a way to eliminate the negative effect of these links and improve the accuracy of the *k*-shell method in determining network core structure.

### Defining the diffusion importance for links

We define the diffusion importance of links in the following way. Consider an edge *e*_*ij*_. When a disease spreads along it, there are two possible directions. In one direction, the disease origins from node *i* and spreads along *e*_*ij*_ to node *j*, and then spreads to the other parts of the network through node *j*. We record the number of links of node *j* connecting outside the nearest neighborhood of node *i* as *n*_*i*→*j*_. In the other direction, the disease origins from node *j* and spreads along *e*_*ji*_ (the same edge as *e*_*ij*_ because it is undirected edge) to node *i*, and then spreads through node *i* to the other parts of the network. We record the number of links of node *i* connecting outside the nearest neighborhood of node *j* as *n*_*j*→*i*_. Then the diffusion importance of edge *e*_*ij*_ is defined as
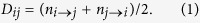
This value quantifies the average potential influence of an edge in both directions. Let’s take edge *e*_*ij*_ in Fig. 1 as an example to calculate the diffusion importance. In Fig. 1(a), *n*_*i*→*j*_ = 0, which is the number of links of node *j* that connect outside the neighborhood of node *i*. At the same time, *n*_*j*→*i*_ = 0, which reflects that node *i* has no link connecting to nodes that are not in the neighborhood of node *j*. Thus the *D*_*ij*_ = 0. In Fig. 1(b), *n*_*i*→*j*_ = 3, *n*_*j*→*i*_ = 2, and thus *D*_*ij*_ = 2.5. In this way, we can calculate the diffusion importance for all edges in the network. When each edge is assigned a diffusion importance, the unweighted graph becomes weighted graph. The weight on edge contains the information of the potential spreading coverage when a disease spreads along the edge. For a general discussion of the weighted network is not in the scope of this paper, which we will explore in the future. Here, we concentrate on identifying links that are less important in the spreading process but lead to form a locally densely connected local structure, which results in the failure of the *k*-shell method to accurately determine the coreness of nodes in spreading dynamics.

### Filtering out redundant links and applying the *k*-shell method to obtain a new coreness for nodes

From the analysis of Fig. 1, we come to the idea that links with low diffusion importance are redundant links, which contribute much to a densely connected local structure and a high *k*_*s*_ for nodes but have a limited diffusion influence. We set a redundant threshold *D*_*thr*_ to determine redundant links. If *D*_*ij*_ < *D*_*thr*_, edge *e*_*ij*_ is considered as a redundant link. If we use *G* = {*V*, *E*} to represent a graph, where *V* is the set of nodes and *E* is the set of edges, then the residual network that is obtained by filtering out redundant links is represented as *G*′ = {*V*′, *E*′}, where *V*′ = *V* and 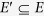. If all edges in the network have a *D*_*ij*_ ≥ *D*_*thr*_, then *E*′ = *E*.

We first apply the *k*-shell decomposition to the original networks and obtain the coreness for each node, recorded as 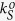. Then we identify and filter out the redundant links. Given that filtering out too many edges may destruct the main structure of the network, the *D*_*thr*_ should not be too large which will lead to a large proportion of links being identified as redundant links. Meanwhile, the *D*_*thr*_ should not be too small because the redundant links that contribute much to the local densely connected structure may have a diffusion importance greater than 0 but are still not so important in a spreading process. We adopt a diffusion threshold of *D*_*ij*_ = 2. For a discussion of the diffusion threshold, please see SI for details. In this case, edges with *D*_*ij*_ ≥ 2 are remained in *G*′. We apply the *k*-shell method to *G*′ and obtain a renewed coreness for each node, recorded as 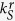. We use the imprecision function, which is initially proposed by Kitsak *et al.*^29^ and modified by Liu *et al.*^39^, to compare the accuracy of 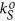 and 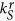 in determine node coreness in the network. The imprecision function is defined as
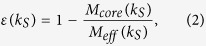
where *k*_*s*_ is the shell index ranging from 0 (for isolated nodes in the residual network) to the maximum *k*_*s*_ value in the network. *M*_*core*_(*k*_*s*_) is the average spreading efficiency of nodes with coreness *k*_*S′*_ ≥ *k*_*S*_ (nodes in *k*_*s*_-core), and *M*_*eff*_(*k*_*s*_) is the average spreading efficiency of *n* nodes with the highest spreading efficiency, where *n* equals to the number of nodes in *k*_*s*_-core. This function quantifies how close to the optimal spreading is the average spreading of nodes in *k*_*s*_-core. A small *ε*(*k*_*s*_) value means nodes identified as in core shells have a correspondingly high spreading efficiency.

**Figure 3:**
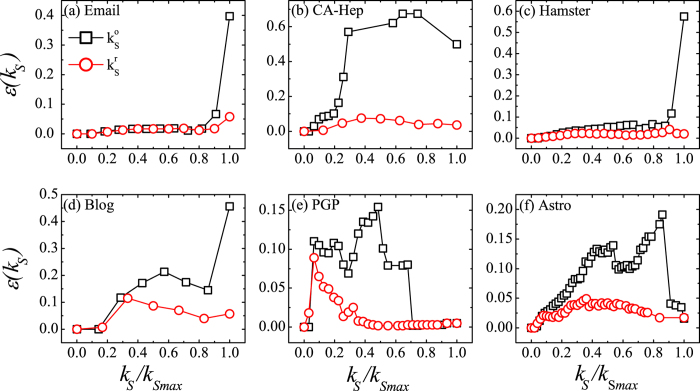
The imprecision of 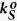 and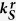 as a function of shell index. 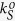
 is the coreness obtained from the original network, and 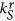 is the coreness obtained from the residual network. Shell index *k*_*s*_ ranges from 0 to *k*_*Smax*_ and is normalized by *k*_*Smax*_. The imprecision of 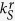 is obviously smaller than that of 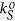.

In Fig. 3 we compare the imprecision of 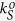 and 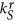. The number of shells may be different for the original graph *G* and the residual graph *G*′, so we normalized the shell index *k*_*s*_ by the maximum shell index *k*_*Smax*_ in *G* and *G*′ respectively. The imprecision based on 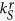 is in general obviously lower than the imprecision based on 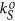. For the networks of Email, CA-Hep, Hamster and Blog, the imprecision of 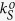 is high for large values of *k*_*S*_, close to or above 0.4. This means that in these networks nodes identified as core by the 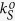 are in fact not very influential in a spreading process. In the network of PGP and Astro, there are sudden jumps in 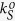 imprecision, which correspond to the locally densely connected structure that does not exist in the innermost core but exists in some outer shells of the network^39^. On the contrary, when the 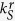 is used to determine node coreness, a much lower imprecision is obtained. In all the studied real-world networks, the absolute value of the imprecision based on 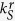 is close to or smaller than 0.1. This means that the 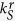 is a good indicator of spreading efficiency. After removing the redundant links with low *D*_*ij*_ values, the accuracy of the *k*-shell method in determining cores is greatly improved.

In many cases, people are more interested in top ranked nodes, which correspond to leaders in the society. We rank nodes by their coreness 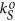 and 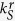 respectively and compare the accuracy of coreness in identifying the most influential spreaders. Results show that the coreness obtained from the residual network is much more accurate than the original coreness in identifying the most influential spreaders. See Fig. S3 in SI for more details.

**Figure 4:**
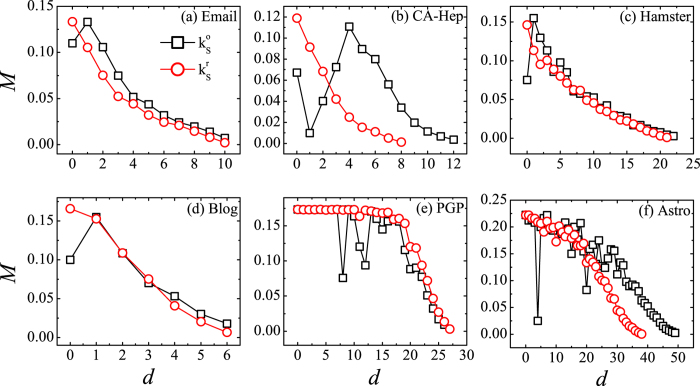
Spreading efficiency of a shell and its distance from the innermost core. 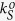
 is the coreness obtained from the original network, and 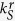 is the coreness obtained from the residual network. *d* is the distance from the innermost core. *d* = 0 corresponds to the innermost core.

Then we focus on the spreading efficiency of shells. A good partition of the network is supposed to display a concordant trend between the shell index obtained from network topology and the spreading efficiency of that shell. One would expect that shells with large *k*_*S*_ should have a higher spreading efficiency than shells with small *k*_*S*_. We plot the spreading efficiency *M* of each shell (expressed as the distance *d* of a shell from the innermost core), where the spreading efficiency of a shell is the average spreading efficiency of nodes in that shell. As shown in Fig. 4, the spreading efficiency of shells is in general decreasing monotonically with the increase of distance from the innermost core in all studied networks when 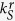 is used. In the networks of Email, CA-Hep and Blog, the spreading efficiency of each shell and its coreness 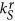 is completely concordant. A large 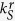 indicates a higher spreading efficiency of the shell. In the networks of Hamster, PGP and Astro, the spreading efficiency and its coreness 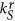 are concordant in most shells. There are a limited number of shells where the trend is not so monotonic, however the fluctuation in spreading efficiency is relatively small compared to that of the 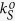. As for the 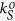, the trend is not as monotonic as 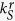. In other words, the coreness obtained from the residual network predicts the spreading efficiency much more accurately than the original one.

### Comparing with random deletion and other way of targeted removing of links

**Figure 5:**
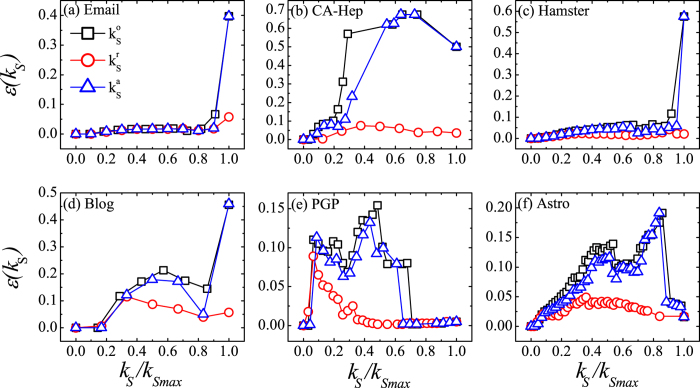
The imprecision of 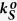, 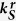 and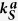 as a function of shell index. 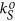
 is the coreness obtained from the original network, 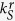 is the coreness obtained from the residual network and 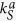 is the coreness obtained from the network after random deletion of edges. Shell index *k*_*s*_ ranges from 0 to *k*_*Smax*_ and is normalized by *k*_*Smax*_. The imprecision of 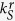 is obviously smaller than that of 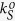 and 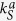.

Our way of removing redundant links obviously improves the accuracy of the *k*-shell method in determining the influence of nodes in a spreading. Now we compare the effectiveness of our way of targeting the redundant links with random deletion, as well as targeting links whose importance is determined by the degree of nodes on its two ends. To compare with random deletion, we randomly select a set of edges and delete them from the network. The number of edges that is to be deleted is the same as the number of identified redundant links. Then we apply the *k*-shell decomposition to the residual network and obtain a *k*_*S*_ for each node. We realize the random deletion for 50 times and average the *k*_*S*_ obtained at each realization as coreness for node *i*, which we record as 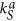 to represent random or arbitrary deletion. A comparison of the imprecision as a function of shell index are shown in Fig. 5. In most cases, the imprecision of 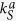 is very close to that of the 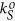 obtained from the original network, and is obviously higher than the imprecision of 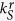 obtained from the residual network. This implies that the core-like groups still exist in the residual network after random deletion of links. Although the imprecision of 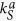 is slightly improved in some networks, we think it is because that when the links are selected randomly, there is a chance that a redundant link is selected.

A widely used way of determining edge importance is considering the degree of nodes on its two ends. The weight (also the importance) of an edge *e*_*ij*_ is proportional to the product of *k*_*i*_ and *k*_*j*_ as*w*_*ij*_ = (*k*_*i*_ *k*_*j*_)^*θ*^, where *k*_*i*_ and *k*_*j*_ are the degree of node *i* and node *j* respectively^45^,^46^,^47^ and *θ* is a tunable parameter. This measure is also strongly correlated with the betweenness centrality of an edge^48^. We use a parameter *θ* = 1 to determine the edge importance, and remove the edges of the smallest weight from the network to see its effect on the *k*-shell method. The number of edges removed is the same as the number of redundant links identified. We find that the imprecision of coreness 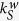 obtained from the residual network in this way is almost the same as the original 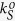, as shown in Fig. S4 in SI.

The above analysis implies us two points. First, our way of identifying and removing the redundant links is effective in improving the accuracy of *k*-shell method in profiling the core structure of the network in a spreading dynamics. Second, the *k*-shell index has a robustness against random failure, which is consistent with the result in Ref. 29. In that work, authors pointed out that the *k*-shell method is robust under random deletion of even up to 50% of the edges, which means the relative ranking of the *k*_*S*_ value for the same nodes in the original network and the network after random deletion are almost the same.

## Discussion

Profiling the network hierarchical structure is very important in understanding the behaviors on it. The *k*-shell decomposition is a basic method to describe network structure and identify core areas that is used in many fields of science. We study the *k*-core structure of real-world networks and the spreading process on it. We find that the accuracy of the *k*-shell method in identifying influential spreaders is impacted by the locally densely connected group in the network, which corresponds to real-world scenarios such as extensive communication and cooperation within a small group or community. Based on in-depth analysis of network local structure and motivated by research advances in core-periphery structure, we realize that the links of nodes contribute differently to the affected population in a spreading process. For the first time we define a diffusion importance for each link in the network based on its potential influence in a spreading process. By filtering out redundant links and then applying the *k*-shell decomposition to the residual graph, we get a renewed coreness for nodes. Experimental results show that this renewed coreness is much more accurate in determining the spreading influence of node from the core to the periphery. Specifically speaking, the imprecision of coreness in identifying influential spreaders is greatly reduced. Nodes with high renewed coreness are in general have a higher spreading efficiency than nodes with low renewed coreness.

There are many algorithms using the *k*_*S*_ index as a global importance of nodes and ranking nodes. Among them, the iterative resource allocating (IRA) algorithm^33^ greatly enhance the accuracy of centrality measures in ranking node influence by iteratively relocating sources to each node based on the centrality of its neighbors (see Methods for details). After iteration, the resource of a node will be stable and is used to rank node of its spreading influence. As above, we filter out the redundant links of *G* and apply the *k*-shell decomposition to the residual graph *G*′ to obtain a 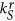 and then implement the IRA algorithm on *G*′. We find that the ranking accuracy is greatly improved, as shown in Fig. S5. The effectiveness of our method in another ranking algorithm, which defines a neighborhood coreness *C*_*nc*_ of node *i* as 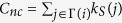 34, where Γ(*i*) is the set of neighbors of node *i* and *k*_*s*_(*j*) is the coreness of node *j*, is shown in SI Fig. S6. We still come to a great improvement in the ranking accuracy.

As our way of filtering out redundant links works well for networks with locally densely connected structure, one may ask the performance of 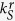 on networks with no such local structure. For the networks of Router, Emailcontact and AS listed in Table 1, in which there is no core-like group and the *k*-shell method works well on the original network, we find that by filtering out redundant links, the performance of 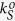 and 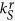 are nearly exactly the same. The imprecision of 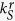 is very low, and high shells always have a high spreading efficiency. This implies that there is no negative effect on the *k*-shell method on networks where it works well. We present the coreness imprecision as a function of shell index and percentage of nodes *p* in SI Fig. S7 and S8 respectively, as well as the spreading efficiency of each shell in Fig. S9. It is again due to the robustness of the *k*-shell method. This feature is meaningful in that our way of filtering out redundant links will greatly improve the accuracy of the *k*-shell method in networks where it doesn’t work well while at the same time doesn’t impact its performance in networks where it already works well. We also test the effects of filtering out redundant links on other centrality measures such as degree centrality, betweenness centrality and eigenvector centrality in ranking node’s spreading influence. Results show that the ranking performance of the centrality obtained from the residual network remains very close to the centrality obtained from the original network. This means the redundant links have little influence on these centrality measures, which is a proof of the redundancy of these links.

Many real-world networks are fractal, which means a topological self-similarity at all length scales, such as the world-wide web, actor collaboration network, protein-protein interaction network and cellular network^49^. An important feature of the fractal network is the disassortativity between hub nodes. Hubs are dispersed making the network more robustness against malicious attack^50^. When applying our method to the fractal network, the links that connect the hub node and the non-hub node will be assigned a relatively large diffusion importance because of the asymmetry of degree on its two ends, thus the number of identified redundant links between hub nodes may be relatively small in fractal networks. In this case, the improvement in identifying the most influential spreaders by our method may be less obvious. Meanwhile, the fractal network is featured by modularity and self-similarity between modules^51^. Modules with local hubs are connected by weak ties^52^. The weak ties between modules may be more important than weak ties within the module in a diffusion process. How to distinguish their influence and define the diffusion importance need to be further studied.

As we use the SIR model to simulate the spreading process, a challenging question is how the algorithm will work when considering real diffusion dynamics, which is much more complex than the model dynamics. When considering information diffusion on Twitter, for example, the attributes of the node (such as its activity level, the social role of whether a mass media, a celebrity or an ordinary user, levels of expertise on various fields, and the biological limits of maintaining stable social relationship) and the tweets itself (such as the topics and spanning time) will largely influence the dynamics^53^,^54^. In addition, the flow of information is directed from the followed one to the follower, and the influence of the information origin is measured by either the number of retweets or mentions^55^. In fact some researchers have addressed the problem of validating the k-shell method in diffusion process on Twitter and find that the *k*_*S*_ index is a good predictor of spreading influence^12^,^32^. However, in behavior spreading, such as the adoption of innovation^56^ and health behavior^57^, the social reinforcement effect of multiple adoption from neighbors will increase the probability of a user to adopt and spread the behavior. In this case, the spreading in a community will be promoted when there are redundant links. The performance of our way in finding good spreaders is worthy of further study when considering different real diffusion processes.

The identification of redundant links gives us implication that redundancy has an effect on the analysis of network structure. While we only concentrate on its effectiveness in *k*-shell method and from the perspective of SIR spreading dynamics, the influence of redundant links on other network analysis remains unexplored. This proposes two challenges. First, we need to decide which structural features of the network are affected much by redundant links, such as the community structure. Second, how to define the importance of links in the network may depend on the real diffusion dynamics on it, such as the rumor spreading, behavior spreading and information diffusion. In addition, while our way of determining the redundant threshold *D*_*thr*_ is obtained from simulation experiments, a parameter-free way of identifying the redundant links is worthy of further explore.

## Methods

### The *k*-shell decomposition

The algorithm starts by removing all nodes with degree *k* = 1. After removing all nodes with *k* = 1, there may appear some nodes with only one link left. We iteratively remove these nodes until there is no node left with *k* = 1. The removed nodes are assigned with an index *k*_*S*_ = 1 and are considered in the 1-shell. In a similar way, nodes with degree *k* ≤ 2 are iteratively removed and assigned an index *k*_*S*_ = 2. This pruning process continues removing higher shells until all nodes are removed. Isolated nodes are assigned an index *k*_*S*_ = 0. As a result, each node is assigned a *k*_*S*_ index, and the network can be viewed as a hierarchical structure from the innermost shell to the periphery shell.

### Identify core-like groups in real-world networks

The link entropy of a shell with index *k*_*S*_ is defined^39^ as
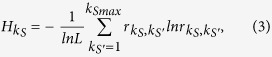
where 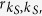 is the average link strength of nodes in the *k*_*S*_-shell to the *k*_*S′*_-shell and *L* is the number of shells in the network. The link strength of node *i* to the *k*_*S′*_-shell is the ratio of the number of links originating from node *i* to the *k*_*S′*_-shell to the total number of links of node *i*. The shells which have a relatively low entropy compared with its adjacent shells are usually locally connected core-like groups.

### SIR model

We use the susceptible-infected-recovered (SIR) spreading model to simulate the spreading process on networks and obtain the spreading efficiency for each node. In the model, a node has three possible states: *S* (susceptible), *I* (infected) and *R* (recovered). Susceptible individual become infected with probability *λ* if it is contacted by an infected neighbor. Infected nodes contact their neighbors and then change to recovered state with probability *μ*. For generality we set *μ* = 1. Recovered nodes will neither be infected nor infect others any more, and they remain the *R* state until the spreading stops. Initially, a single node is infected and all others are susceptible. Then the disease spreads from the seed node to the others through links. The spreading process stops when there is no infected node in the network. The proportion of recovered nodes *M* when spreading stops is considered as the spreading capability, or spreading efficiency, of the origin node. We realize the spreading process for 100 times and take the average spreading efficiency of a node as its spreading efficiency.

As we have discovered that the infection probability will not change the relative spreading efficiency of nodes^39^, in this paper we chose an infection probability *λ* > *λ*_*c*_, where 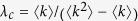 is the epidemic threshold determined from the heterogenous mean-field method^58^. Under the infection probability of *λ*, the final infected population *M* is above 0 and reaches a finite but small fraction of the network size for most nodes as the spreading origin, in the range of 1%–20%^29^.

### Ranking algorithm of IRA

This algorithm considers that the spreading influence of a node is determined by both its centrality and its neighbor’s centrality^33^. In an iterative resource allocation process, the resource of nodes is distributed to its neighbors according to their centrality. The resource node *i* receive is
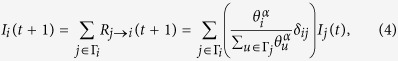
where *R*_*j*→*i*_(*t* + 1) is the amount of resource distributed from node *j* to node *i* at time *t* + 1, Γ_*i*_ is the sets of node *i*'s neighbors. *θ*_*i*_ is the centrality of node *i*, and *α* is a tunable parameter to adjust the influence of centrality. *u* belongs to the neighborhood Γ_*j*_ of node *j. δ*_*ij*_ = 1 if there is a link between node *i* and node *j*, otherwise *δ*_*ij*_ = 0. *I*_*j*_(*t*) is the resource hold by node *j* at time step t. Initially, each node has a unit resource. After iterations the resource distributed to each node will be stable, and the final resource of nodes are used to rank their spreading influence. The coreness centrality *k*_*S*_ is used here, and *α* is set to 1.

### Data sets

The real-world networks studied in the paper are: (1) Email (e-mail network of University at Rovira i Virgili, URV)^59^; (2) CA-Hep (Giant connected component of collaboration network of arxiv in high-energy physics theory)^60^; (3) Hamster (friendships and family links between users of the website hamsterster.com)^61^; (4) Blog (the communication relationships between owners of blogs on the MSN (Windows Live) Spaces website)^62^; (5) PGP (an encrypted communication network)^63^; (6) Astro physics (collaboration network of astrophysics scientists)^64^; (7) Router (the router level topology of the Internet, collected by the Rocketfuel Project)^65^; (8) Emailcontact (Email contacts at Computer Science Department of University College London)^29^; (9) AS (Internet at the autonomous system level)^66^.

## Additional Information

**How to cite this article**: Liu, Y. *et al.* Improving the accuracy of the *k*-shell method by removing redundant links: From a perspective of spreading dynamics. *Sci. Rep.* **5**, 13172; doi: 10.1038/srep13172 (2015).

## Supplementary Material

Supplementary Information

## Figures and Tables

**Table 1:** Properties of the real-world networks studied in this work.

Network	*N*	*E*	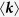	*k*_*max*_	*H*_*k*_	*r*	*C*	*k*_*Smax*_	λ_*c*_	λ
Email	1133	5451	9.6	71	1.942	0.078	0.220	11	0.06	0.08
CA-Hep	8638	24806	5.7	65	2.261	0.239	0.482	31	0.08	0.12
Hamster	2000	16097	16.1	273	2.719	0.023	0.540	24	0.02	0.04
Blog	3982	6803	3.4	189	4.038	−0.133	0.284	7	0.08	0.27
PGP	10680	24340	4.6	206	4.153	0.240	0.266	31	0.06	0.19
Astro	14845	119652	16.1	360	2.820	0.228	0.670	56	0.02	0.05
Router	5022	6258	2.5	106	5.503	−0.138	0.012	7	0.08	0.27
Emailcontact	12625	20362	3.2	576	34.249	−0.387	0.109	23	0.01	0.10
AS	22963	48436	4.2	2390	61.978	−0.198	0.230	25	0.004	0.13

Structural properties include number of nodes (*N*), number of edges (*E*), average degree 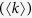, maximum degree (*k*_*max*_), degree heterogeneity (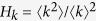), degree assortativity (*r*), clustering coefficient (*C*), maximum *k*_*s*_ index (*k*_*Smax*_), epidemic threshold (λ_***c***_), infection probability used in the SIR spreading in the main text (λ) (see Method for details). For the first six networks, there exist core-like groups. While for the last three networks, there is no core-like group in the network, which we will discuss in the last part.
