# Supplementary material for: Improving the accuracy of the k-shell method by removing redundant links: From a perspective of spreading dynamics
Source: Sci Rep. 2015 Aug 17;5:13172. doi: 10.1038/srep13172 (PMC4538382; doi:10.1038/srep13172)
Supplement: Supplementary Information [file srep13172-s1.pdf]

Supporting Information for

Improving the accuracy of the k-shell method by removing redundant links: From a perspective of spreading dynamics

Ying Liu, Ming Tang, Tao Zhou and Younghae Do

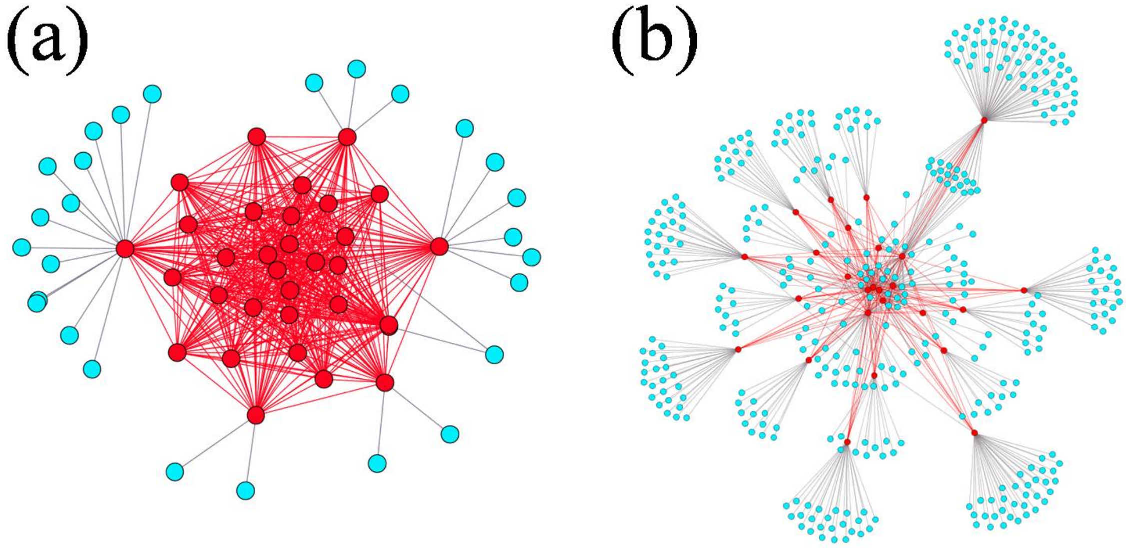

Figure S1. Visualization of the neighborhood of the innermost core of CA-Hep and Router. The core neighborhood of two real-world networks are visualised by Gephi version 0.8.2. Red nodes are the innermost core nodes and the blue nodes are neighbors of the core nodes that are not in the core. Links that connect the core nodes and the core nodes to their neighbors are shown. (a) Core neighborhood of CA-Hep. The innermost core of CA-Hep is composed of 32 nodes with k-shell index 31. For most of the core nodes, their links are limited within the core. Only five core nodes have a very few number of links that connect to nodes outside the core. This is a core-like group, but has a maximal k_S index in the network. (b) Core neighborhood of Router. The innermost core of Router is composed of 26 nodes with a maximal k-shell index 7. Each of the core nodes has a large amount of links that connect to nodes outside the core.

We demonstrate the influence of D_{thr} on the imprecision of coreness k_S^r. We use the imprecision function proposed by Kitsak *et. al* as

$$\varepsilon(p) = 1 - \frac{M_{core}(p)}{M_{eff}(p)}, \tag{1}$$

where $M_{core}(p)$ and $M_{eff}(p)$ are the average spreading efficiency of pN nodes with the highest coreness and largest spreading efficiency, respectively, and p is the fraction of network size N. The smaller the imprecision, the more accurate measure of coreness to predict node's spreading efficiency . When more than one nodes have a same k_S^r at certain p, a node is selected randomly. We choose four p values, that are $p = 0.01$, $p = 0.05$, $p = 0.1$, $p = 0.2$. The imprecision is shown in Fig. S2. $D_{thr} = 0$ corresponds to the original network, since all edges have

a diffusion importance $D_{thr} \geq 0$. For the networks of Email, CA-Hep and Astro, the imprecision remains stable with the increase of D_{thr} when $D_{thr} \geq 0.5$. For the networks of Blog and PGP, the imprecision decreases with the increase of D_{thr}. For the network Hamster, the imprecision is stable when $D_{thr} \geq 0.5$, except for $p = 0.01$ where there is a decrease at $D_{thr} = 2.0$. For the networks of Router, Emailcontact and AS, the imprecision keeps almost unchanged for all D_{thr}. As the k-shell method has a good robustness, we use a threshold $D_{thr} = 2$. In table S1 we list the proportion of links that are identified as redundant links under different threshold D_{thr}. It can be seen that within the range $D_{thr} \leq 3$, the percentage of identified redundant links is basically within 30%, except PGP for 31.4%.

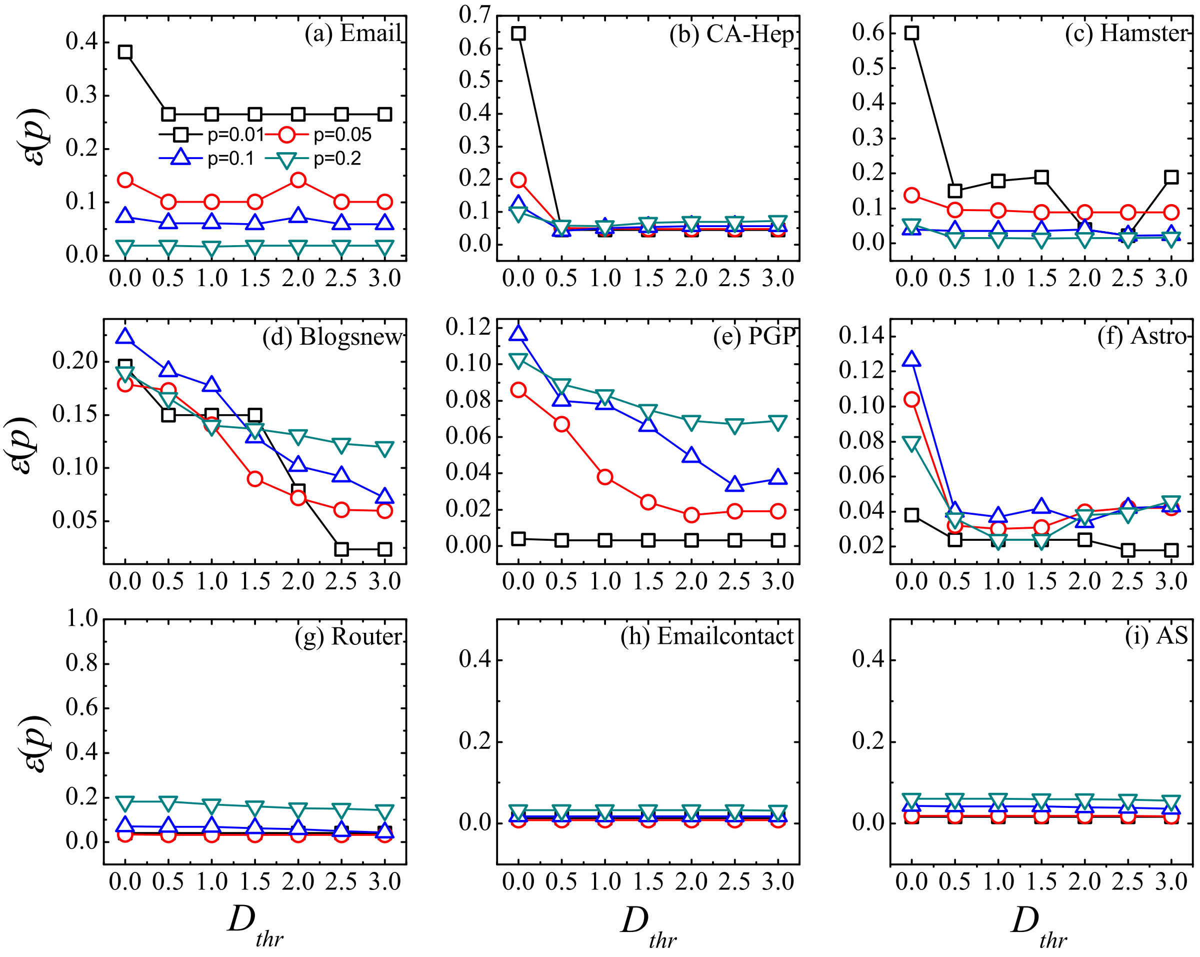

Figure S2. Imprecision of k_S^r as a function of D_{thr} for nine real-world networks. k_S^r is the coreness obtained from the residual network. p is the proportion of nodes calculated. D_{thr} is the threshold for identifying redundant links.

Table S1. Percentage of redundant links under different diffusion threshold D_{thr}. $D_{thr} = 0.5$ means an edge e_{ij} with diffusion importance $D_{ij} < 0.5$ is identified as a redundant link.

Network	Email	CA-Hep	Hamster	Blog	PGP	Astro	Router	Emailcontact	AS
$D_{thr} = 0.5$	0.04%	6.6%	4.1%	2.7%	3.1%	8.9%	0.1%	0	0.02%
$D_{thr} = 1.0$	0.2%	9.8%	6.2%	7.0%	8.2%	10.5%	13.2%	0.05%	0.4%
$D_{thr} = 1.5$	0.5%	13.4%	7.6%	11.8%	14.7%	12.1%	18.7%	0.2%	1.5%
$D_{thr} = 2.0$	1.1%	17.0%	9.0%	17.0%	20.7%	14.3%	22.1%	0.3%	2.9%
$D_{thr} = 2.5$	1.7%	20.8%	10.7%	21.6%	26.2%	16.2%	24.8%	0.4%	4.4%
$D_{thr} = 3.0$	2.8%	24.6%	11.7%	26.0%	31.4%	18.2%	27.3%	0.5%	5.8%

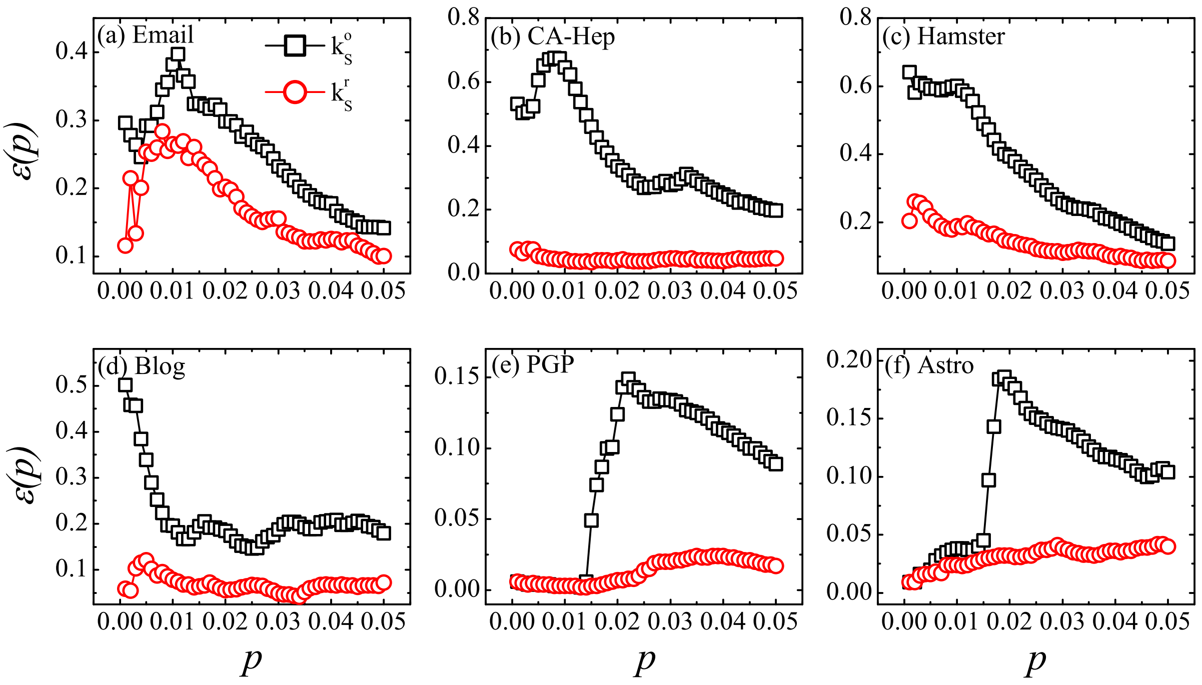

Figure S3. The imprecision of k_S^o and k_S^r as a function of p for six real-world networks . k_S^o is the coreness obtained from the original network, and k_S^r is the coreness obtained from the residual network. p is the proportion of top ranked nodes under consideration, and p ranges from 0.001 to 0.05. The imprecision of k_S^r is obviously smaller than that of k_S^o. This means that in identifying the most influential spreaders, the k_S^r is much more accurate than the k_S^o.

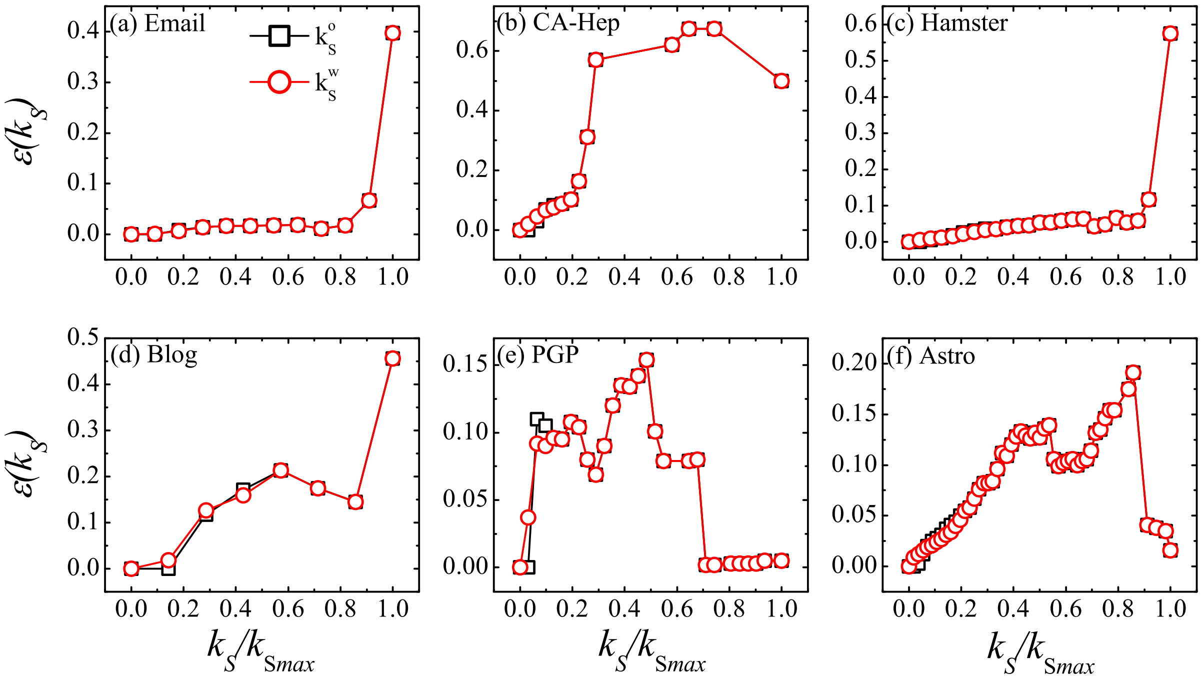

Figure S4. The imprecision of k_S^o and k_S^w as a function of shell index for six real-world networks. k_S^o is the coreness obtained from the original network, and k_S^w is the coreness obtained from the network after removing links of small weight. The imprecisions of k_S^o and k_S^w are almost the same. k_S ranges from 0 to k_{Smax} and is normalized by k_{Smax}.

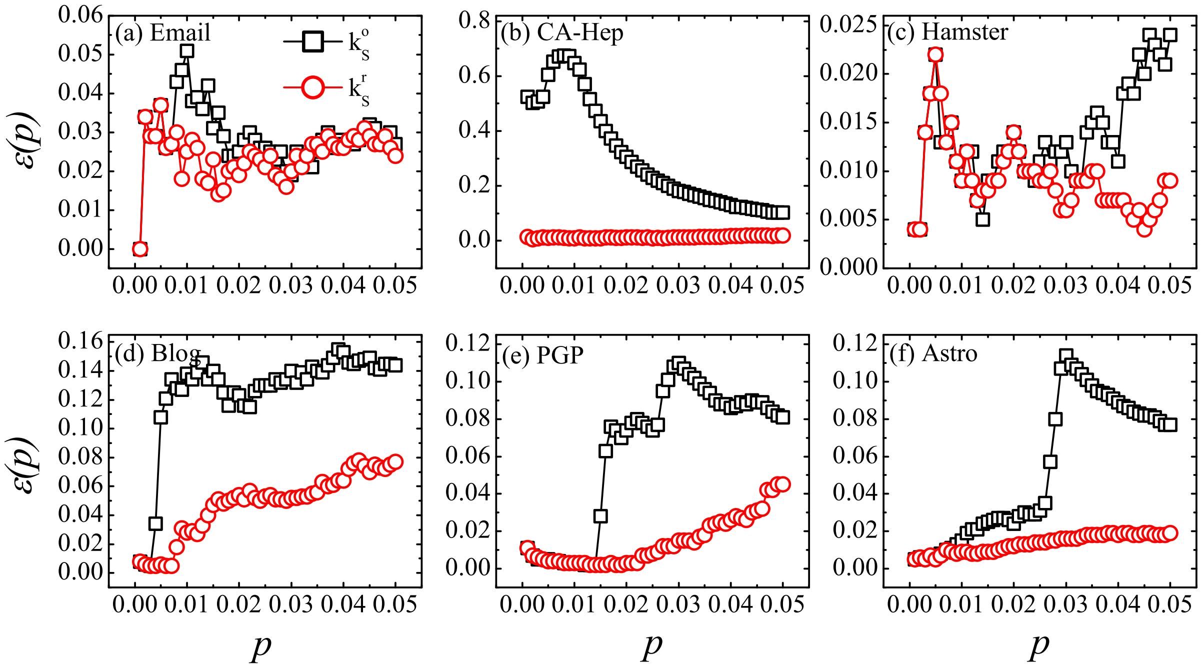

Figure S5. The imprecision of IRA based on k_S^o and k_S^r as a function of p for six real-world networks. k_S^o is the coreness obtained from the original network, and k_S^r is the coreness obtained from the residual network. p is the proportion of top ranked nodes under consideration, and p ranges from 0.001 to 0.05. In all studied networks, the ranking imprecision based on k_S^r is obviously lower than that of k_S^o, and is less than 0.1 for all p in the demonstrated range. There are only a few exceptions that the imprecision based on k_S^o is smaller than that of k_S^r in Email and Hamster networks, but the difference is very small.

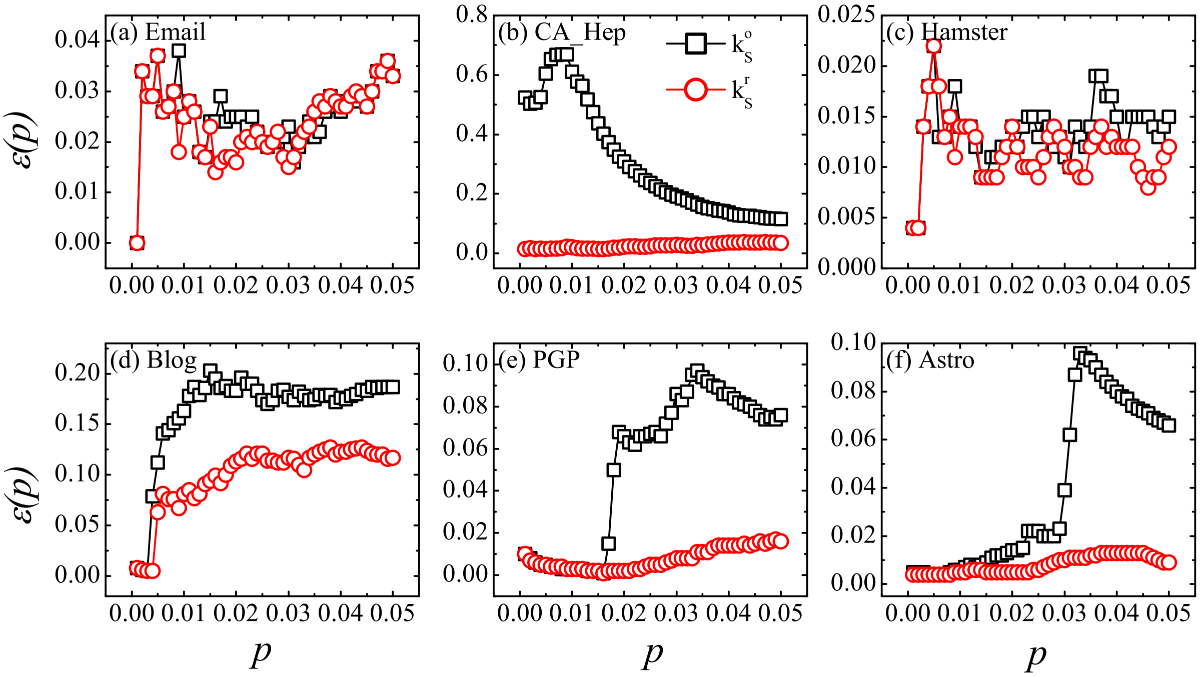

Figure S6. The imprecision of C_{nc} based on k_S^o and k_S^r as a function of p for six real-world networks. k_S^o is the coreness obtained from the original network, and k_S^r is the coreness obtained from the residual network. By using k_S^r, the ranking accuracy of the neighborhood coreness C_{nc} is greatly enhanced.

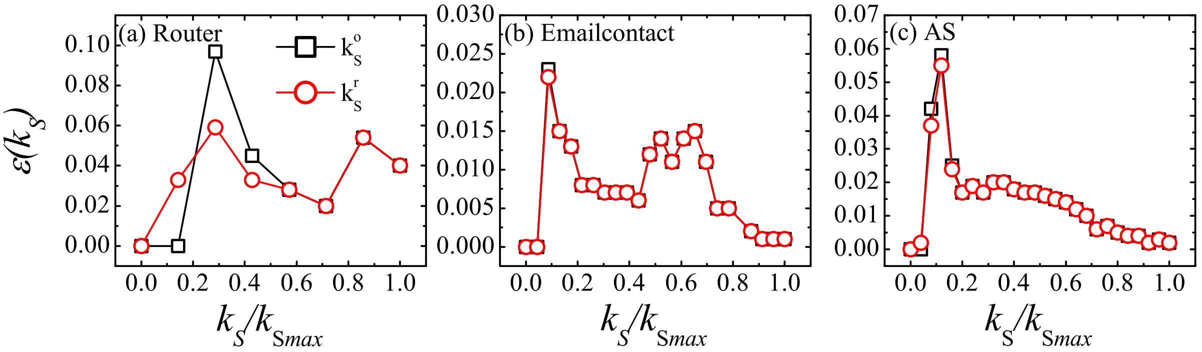

Figure S7. The imprecision of k_S^o and k_S^r as a function of shell index k_S for three real-world networks with no core-like groups. k_S ranges from 0 to k_{Smax} and is normalized by k_{Smax}. The imprecision of k_S^r is very close to that of k_S^o. In Router network, at the point $k_S/k_{Smax} \approx 0.14$, the shell index is 1 ($k_{Smax} = 7$). For the original network G, $1 - core$ contains all nodes in the network, and thus the $\varepsilon_{k_S^o}(1) = 0$. As for G', there are some nodes with $k_S = 0$, which are isolated nodes during the edge removing process, and thus the $\varepsilon_{k_S^r}(1) > 0$.

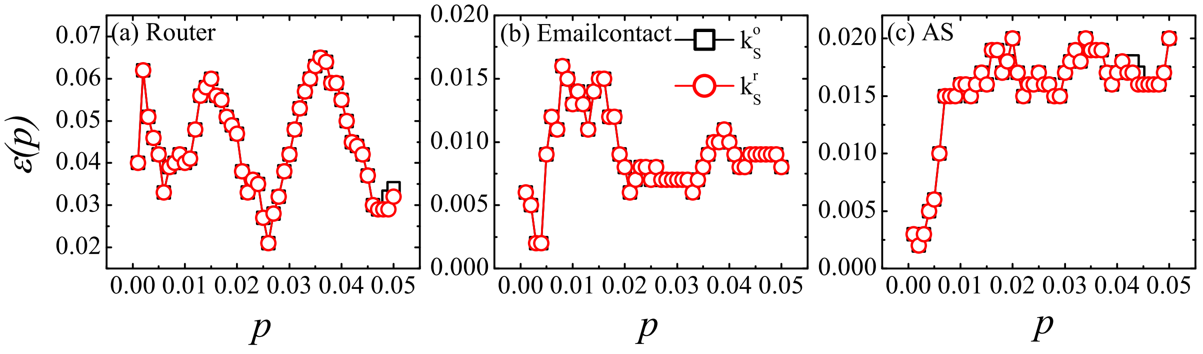

Figure S8. The imprecision of k_S^o and k_S^r as a function of p for three real-world networks with no core-like groups. k_S^o is the coreness obtained from the original network, and k_S^r is the coreness obtained from the residual network. p ranges from 0.001 to 0.05. In all the three networks, the imprecision of k_S^o and k_S^r are almost the same.

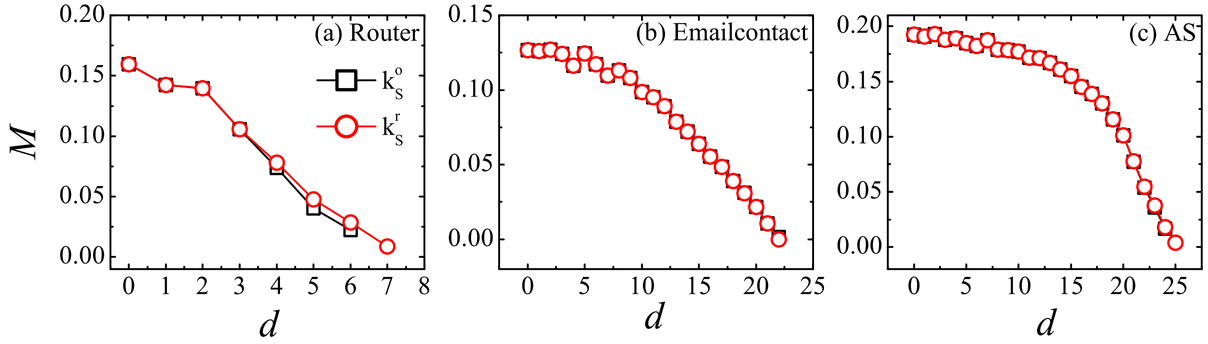

Figure S9. Spreading efficiency of each shell as a function of d for three real-world networks with no core-like groups. k_S^o is the coreness obtained from the original network, and k_S^r is the coreness obtained from the residual network. M is the spreading efficiency of each shell, which is the average spreading efficiency of nodes in that shell. d is the distance of a shell from the innermost core shell. The monotonic trend of d with M are almost the same for both k_S^o and k_S^r.
